# Okra (*Abelmoschus esculentus* L.) as a Potential Functional Food Source of Mucilage and Bioactive Compounds with Technological Applications and Health Benefits

**DOI:** 10.3390/plants10081683

**Published:** 2021-08-16

**Authors:** Thamires Lacerda Dantas, Flávia Carolina Alonso Buriti, Eliane Rolim Florentino

**Affiliations:** 1 Programa de Pós-Graduação em Ciências Farmacêuticas, Universidade Estadual da Paraíba, Campina Grande 58429-600, PB, Brazil; thamires.lacerda.dantas@gmail.com; 2 Núcleo de Pesquisa e Extensão em Alimentos, Universidade Estadual da Paraíba, Campina Grande 58109-790, PB, Brazil

**Keywords:** *Abelmoschus esculentus*, okra, functional food, polysaccharides, gum, mucilage

## Abstract

*Abelmoschus esculentus* has fruit popularly known as okra and belongs to the *Malvaceae* family. It is commonly used in cooking but also in traditional medicine in the treatment of worms, dysentery, inflammation, and also irritation of the stomach, intestines, and kidneys, as it is a potential functional food. Its mucilage is a highly viscous polysaccharide that is mostly composed of monosaccharides D-galactose, L-rhamnose, and galacturonic acid, as well as proteins and minerals. The functional properties of okra mucilage have been widely studied, mainly for its potential antidiabetic activity; thus, its use as adjuvant or nutraceutical therapy for diabetes is very promising. Due to its rheological properties, it is a potential resource for pharmaceutical and food applications. Okra mucilage can be extracted by several methods, which can directly influence its physicochemical characteristics and biological activity. Features such as low cost, non-toxicity, biocompatibility, and high availability in nature arouse the interest of researchers for the study of okra mucilage. The survey of research on the applications of okra mucilage highlights the importance of using this promising source of bioactive compounds with interesting technological properties. The potential of okra as a functional food, the properties of okra mucilage, and its technological applications are discussed in this review.

## 1. Introduction

*Abelmoschus esculentus* L. is popularly known as okra or lady’s finger. It belongs to the Malvaceae family, native to Africa, and is cultivated in tropical, subtropical, and warm temperate climates in different countries from Africa to Asia, Southern Europe, and America [1,2].

**Figure 1:**
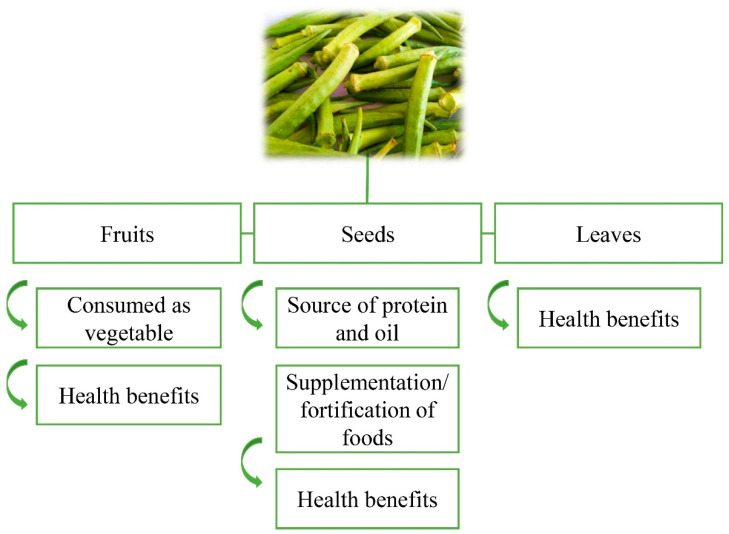
Utilization of fruits, seeds, and leaves from okra. Okra image: authors’ personal archive.

Okra, originating in Ethiopia and propagated in North Africa, the Mediterranean, Arabia, and India is of great economic importance in the subtropical regions of the world [3]. As food, okra can be eaten fresh or cooked and used as an additive in soups, salads, and stews [4]. Okra fruit has a high moisture content, it is rich in nutrients, and it is a great source of vitamins and minerals. Carbohydrates are present in okra mainly in the form of mucilage [5], commonly applied in different industrial segments and for medicinal purposes [6]. The fruits, seeds, and leaves of okra have applications due to their composition and properties (Figure 1).

Okra is a vegetable widely cultivated in the world. It is considered important throughout the tropical and subtropical regions of Africa and Asia, with an annual estimated production of six million tons [7]. In Pakistan, okra crop is grown on an area of 15,081 ha with an annual production of 114,657 tons [8]. Globally, India ranks first in okra production, having an area of 509 ha with an annual production of 6094.9 million tons and productivity of 12 million tonnes/ha [9].

The bioactive potential of okra mucilage and its rheological characteristics have been the subject of several studies and reported in current bibliographic surveys [10,11]. However, the importance of okra as a low-cost functional food still deserves to be more underlined. Functional foods represent an important segment for innovation, as they are intended not only to satisfy hunger and provide human beings with the necessary nutrients but also to prevent nutrition-related illnesses and increase consumers’ physical and mental well-being [12]. The richest sources of compounds with beneficial health effects are vegetables due to their richness in polyphenols, a very efficient source of antioxidants [13]. In this sense, this paper aims to highlight the benefits of this fruit as a low-cost raw material and a promising natural alternative in several areas, including its use as a functional food, particularly the use of okra mucilage as a technological resource with interesting properties for industry use.

## 2. Composition, Cost, and Main Uses of Okra

Several in natura foods are consumed daily in dishes in their raw state or included in their cooked state in dishes and regional preparations; most people are unaware of their potential health benefits [14]. However, industrialized functional foods have added production value, which raises their final cost to consumers. This higher price is in part due to the various health benefits they provide, in part to the production costs, and also in part to the marketing carried out on them by companies to win over consumers, seeking to give greater emphasis so that people keep in mind that they are investing in good health and quality of life [15].

An alternative for the population is to take advantage of the potential of in natura and minimally processed foods, such as fruits, vegetables, cereals, and legumes. Vegetables, such as okra, have functional properties, and, in addition to being produced on a large scale, most of them have a low cost for the consumer.

The functional properties of okra are not just limited to the fruit. Studies show that parts of the plant, such as the leaves, flowers, and seeds, have bioactives in their composition [16,17,18,19]. Fauza et al. [20] highlighted the phenols and flavonoids present in okra fruits as being the main components responsible for their beneficial properties. Assessing the biological activity of okra fruit flour in vivo, a decrease in glucose levels was observed by the same authors.

According to the last agricultural census of the Brazilian Institute of Geography and Statistics (IBGE) [21], the production of okra in Brazil reached 111.967 tons, being grown in 43,341 agricultural establishments, located mainly in the Northeast and Southeast regions. The Brazilian production value of okra corresponded to 2.29% of the total spent on vegetables. The price of okra in Brazil by May 2021 ranged between 3.8 and 7.2 BRL/Kg (0.69–1.29 USD/kg), with the lowest price found in the Northeast and Southeast regions [22].

Okra is a nutrient-rich food and its inclusion in one’s diet can bring many benefits. Dietary fibers are the most abundant macronutrients (8.16 g/100 g fresh weight), followed by carbohydrates (4.86 g/100 g fresh weight) and proteins (3.55 g/100 g fresh weight) [23]. Despite the low-fat content (0.19 g/100 g) and energy (33 kcal/100 g equivalent to 138 kJ/100 g) of okra fruits [24], their seeds contain unsaturated fatty acids, such as linoleic acid, that are essential for human nutrition [5,25]. These seeds are also rich in α-tocopherol and have high levels of minerals, including Ca, K, Cu, Fe, P, Mg, Zn, and Mn [26,27]. 

The protein content of okra is relatively high when compared to other vegetables, concentrating particularly in its seeds [28]. According to Ofori, Tortoe, and Agbenorhevi [29], depending on the okra variety, the protein content of the seed meal can vary between 16.80 and 17.40%. Some authors have reported values above 40% [16,30], which configures okra as an important source of protein. According to Gerrano [31], the difference in the protein content might be due to genetic and environmental conditions prevailing during the growing period of okra pods.

Some studies have used okra in the preparation of flours to enrich foods such as cakes and cookies, obtaining innovative and functional products [32,33,34]. The partial substitution of wheat flour for okra flour has nutritionally enriched the product made by Rindiani and Kumalasari [34], increasing the amount of fiber, since okra flour has 14.21% fiber, while wheat flour has 2.7%. Brito et al. [32] also produced a cake with okra flour and observed a good sensory acceptance of the product, contrary to what was observed by Oliveira et al. [33], who did not obtain acceptable sensory characteristics in the developed product.

## 3. Beneficial Properties of Okra Mucilage to Health and Its Relevance for Considering Okra as a Functional Food

The search for foods enriched in nutrients and bioactives with properties that help the proper functions of the organism with improvement of the consumers’ health reveals the importance of functional foods. In this sense, the definition of a “functional food” is related to its nutritional properties and to the metabolic and physiological effects bringing health benefits. In particular for okra, its mucilage is of valuable importance for considering this vegetable as a potential functional food. Okra mucilage can be consumed in natura and can be considered as a natural functional food itself due to its potential health benefits.

Okra mucilage is a mixture of natural polysaccharides, consisting of the monosaccharides D-galactose, L-rhamnose, and galacturonic acid associated with proteins and minerals [35]. According to Adetuyi and Dada [36], the content of Zn and Ca in the mucilage of okra is higher than the mineral content of the whole okra fruit. More details related to the composition and physicochemical characteristics of okra mucilage are discussed in the next section. Vegetable mucilages have been studied due to their biological activities in humans and animals. The polysaccharides from these mucilages demonstrate some important biological activities, such as immunomodulated and anti-inflammatory [27,37,38,39].

Okra mucilage especially has demonstrated functional health properties through in vitro and in vivo studies, such as antitumor, antioxidant, antimicrobial, hypoglycemic, and antiulcerogenic capacities, as well as the ability to bind cholesterol and bile acids, removing toxins from the liver [40,41,42,43,44,45,46]. Most studies on the functional properties of okra mucilage also focused on its potential biological activity in controlling the biochemical factors of type 2 diabetes [47,48,49,50,51]. Matazu et al. [52] developed a nutraceutical formulation using okra seeds and peels, rich in mucilage, and evaluated its antidiabetic and antioxidant properties. The improvement in the lipid profile, blood glucose levels, and glycated hemoglobin values indicated the effectiveness of the formulation as a potent antidiabetic agent. Therefore, the use of okra mucilage as nutraceutical therapy or adjuvant therapy for the treatment of this disease is very promising [41]. According to Bonciu [13], some vegetables are true natural nutraceuticals, which helps in the treatment of many diseases.

The viscous nature of okra mucilage can greatly increase inhibition of both in vitro glucose trapping in the cell and the absorption of sugar from the intestine. This fact was confirmed by Chukwuma et al. [37], who compared the anti-hyperglycemic activity of amadumbe (*Colocasia esculenta*) and okra mucilage and observed greater inhibition of glucose absorption by okra due to the higher viscosity of its mucilage.

The natural polysaccharides found in plant mucilages have excellent antioxidant activity, which prevents cell damage caused by reactive oxygen species. In addition to acting on the sequestration of free radicals, these polysaccharides can increase the levels of superoxide dismutase (SOD), favoring the antioxidant mechanism [53,54,55,56]. Okra mucilage carbohydrates, especially the pectic polysaccharide fraction WOP-2 (a rhamnogalacturonan I backbone with type II arabinogalactan side chains substituted partly at *O*-4 of rhamnopyranosyl), have antioxidant activity that can assist in decreasing lipid peroxidation reactions that cause the destruction of beta cells [49].

Okra mucilage is also used in traditional medicine to treat gastric irritations. Some of its properties prevent *Helicobacter pylori* from adhering to stomach tissue. In the findings of Messing et al. [57], it was possible to establish a relationship between the dose of the aqueous extract of immature okra fruits and the inhibition of the cell membrane proteins of the microorganism binding to their respective ligands present in the gastric mucosa.

## 4. Composition and Physicochemical Characteristics of Okra Mucilage

Okra mucilage has a viscous appearance attributed mainly to the acidic polysaccharides that it is made of [58]. These polysaccharides are soluble in acidic or alkaline solutions and in hydro alcoholic solutions. When extracted in water, they result in a highly viscous solution [59].

According to Gao et al. [60], who isolated two polysaccharide fractions from okra (AEP-1 and AEP-2, identified as rhamnogalacturonan I and type II arabinogalactan, respectively), these polysaccharides are white flocculent solids that are not soluble in organic solvents, such as ethanol, acetone, chloroform, and n-butanol, but easily soluble in water.

The main components of okra mucilage polysaccharides are mannose, rhamnose, glucuronic acid, glucose, arabinose, galacturonic acid, galactose, and xylose [27,44,60]. Table 1 provides a brief review of the monosaccharide composition of the okra mucilage, as well as the main methods for its extraction and purification.

The molecular weight of okra polysaccharides can be directly related to their bioactive properties. Low molecular weight polysaccharides are more active than high molecular weight polysaccharides, as they have difficulty crossing cell membranes [62]. Nie et al. [45] evaluated the characteristics of the polysaccharides of different cultivars of okra (Lvjian, Klong8, Shuiguo, Taiwanwufu, and Kalong3) and isolated two fractions from each cultivar after chromatographic analysis. For the different cultivars, the molecular weights ranged from 2.76 × 10^3^ kDa to 4.20 × 10^3^ kDa for a polysaccharide fraction and 0.11 × 10^3^ kDa to 0.9 × 10^3^ kDa for another fraction. According to the same authors, the different cultivars did not influence the composition of the polysaccharides but did influence their molecular weight.

Polysaccharides and phenolic compounds are the main bioactive components of okra. In the fruit and its extracts, one can find the phenolic compounds catechin, isoquercitrin, protocatechuic acid, quercetin, quercetin-3-O-gentiobioside, and rutin [63]. In okra mucilage, Nampuak and Tongkhao [64] observed the presence of catechin, epigallocatechin gallate, and quercetin compounds. Mucilages extracted from okra cultivated in different regions of the world may present differences in their biochemical composition, such as in the content of total phenolics [36,37,61,62].

Pectin is the main polysaccharide present in okra mucilage and has been identified as responsible for the viscous aspect of okra extracts [65]. Classified as a complex polysaccharide, pectin is found in plants and consists mainly of α-1,4 chains linked to D-galacturonic acid [66]. Pectic polysaccharides are used in the industrial sector to promote increased viscosity, as well as to act as a stabilizer and a protective colloid in food and beverages [67].

## 5. Extraction Methods

Okra mucilage can be extracted using some techniques that are mostly based on the use of distilled water or organic solvents. The application of heat is also present in certain processes. Farooq, Malviya, and Sharma [68] extracted the mucilage while keeping the okra stirred in distilled water under continuous agitation at 60 °C for approximately 4 h. Sequentially, the mucilage was isolated with the aid of acetone.

Wang et al. [44] used ultrasound-assisted extraction to obtain water-soluble polysaccharides from okra fruits. The ideal conditions for a better performance of the extraction process were the temperature of 59 °C for a time of 30 min using 522 W of ultrasonic power.

Sengkhamparn et al. [69] extracted the material from the cell wall in different conditions, such as hot buffer, chelating agent, dilute alkaline, and concentrated alkaline. Galactose, rhamnose, galacturonic acid, and arabinose were found in the polysaccharide fractions extracted by those methods, except for the concentrated alkaline method. Arabinose was also found in the polysaccharide obtained using the dilute alkali. For the polysaccharide isolated in concentrated alkali, the presence of XXXG-type xyloglucan and 4-methylglucunoxylan was verified.

Yuan et al. [70] demonstrated that the method of extracting okra mucilage can directly influence its physicochemical characteristics and biological activity. In this study, three extraction processes were compared, namely hot water, pressurized water, and microwave-assisted extraction. The extracts obtained showed monosaccharides with molecular weights, intrinsic viscosity, degree of esterification, and different uronic acids. The antioxidant and inhibitory activity of α-amylase and α-glucosidase varied for each extract obtained.

Okra mucilage is easily extracted in an aqueous medium due to the high solubility of its polysaccharides, and the good yield of extraction is one of the advantages of this method. Cahyana and Kam [71] evaluated the influence of some factors such as time, temperature, and the ratio between water and okra fruits on the extraction yield and on antioxidant and anti-α-glucosidase activities. The different treatments used in the extraction did not significantly influence the yield; however, the extract was obtained by soaking the fruit for 12 h at 4–5 °C. The ratio of 1:6 (fruit:water) showed the best antioxidant activity. The influence of factors such as time and temperature on the yield or biological activity of the mucilage polysaccharides depends on the method used for the extraction. Ultrasound-assisted extraction showed excellent extraction yields (9–10%) at temperatures between 55 and 65 °C for 20 to 30 min [33].

## 6. Applications of Okra Mucilage

Mucilages extracted from plant sources have rheological characteristics with potential for use as thickeners and food stabilizers and are well accepted by consumers because they are natural substances [72]. In the pharmaceutical industry, some mucilages can be used as raw materials to produce natural coatings due to constituents such as pectin, galactans, and glucuronic acid [73]. 

**Figure 2:**
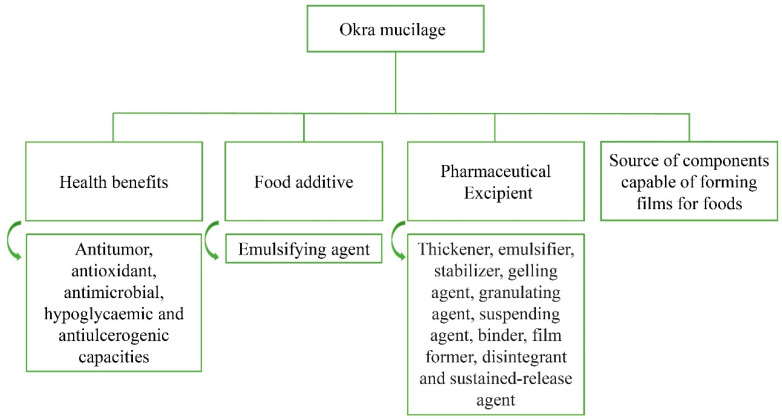
Okra mucilage utilization.

Studies that characterized okra mucilage have reported its physicochemical and rheological properties for pharmaceutical applications and its use as a natural additive in food products and nutraceutical supplements (Figure 2) [74,75,76,77,78]. The main potential applications of okra polysaccharides in different industries are described below.

## 7. Food Technology

Emulsifiers are one of the main classes of additives used in the food industry, and they play an important role in the formation and stability of emulsions, including milks, creams, seasonings, desserts, sauces, and beverages [79,80].

Okra mucilage has been studied as a potential emulsifying agent for food; in addition, its fruit extract is used empirically in traditional cooking to thicken stews and soups. Noorlaila et al. [77] observed this property in the mucilage extracted from okra fruits of different maturation stages from its inclusion in a coconut milk oil–water emulsion system. The results confirmed the possibility of using okra as an emulsifier in the food industry. According to the authors, mucilage can improve the quality of food in terms of stability, texture, and appearance, also acting as gelling agents or texture modifiers.

As texture is a fundamental attribute in food, it is important to search for new agents, mainly from natural sources, that add characteristics to the products, making them more attractive to the consumer. Xu et al. [78] observed that the addition of okra polysaccharides increased the water holding capacity, as well as the firmness and elasticity, in yogurt. Yuenaan, Sajjaanantakul, and Goff [81] also obtained positive results when they included okra polysaccharides in an ice cream formulation. The viscosity increased significantly, and there was a decrease in the growth of ice crystals in the mixture, important factors for a satisfactory sensory perception. Okra mucilage can also act as a partial fat replacer in ice cream without altering the physical and sensory characteristics of the product. This property can improve the nutritional quality of this food product and allows ice creams to be included in low-fat diets [82].

In the food industry, the main natural emulsifier is a lecithin, which is a mixture of phospholipids extracted from animal or vegetable sources [83]. Datsomor et al. [84] evaluated the replacement of lecithin in chocolate with pectin extracted from okra mucilage and observed that the yield obtained for formulations containing 25% okra pectin was higher than that of formulations containing only lecithin. The results indicated that the substitution of lecithin for okra pectin did not affect the sensory characteristics of the chocolate produced.

In addition to the technological properties offered by okra mucilage, this ingredient can also enhance the nutritional value of food products, besides providing the potential as functional foods to them as a result of its bioactive properties, highlighting its antioxidant capacity. Regarding this aspect, Araujo et al. [85] produced a tomato sauce with okra mucilage added with significant amounts of phenolic compounds and high antioxidant activity, and it had good acceptance in relation to important attributes such as uniformity, softness, smoothness, and flavor.

Since biotechnology applied to foods has as one of the most important implications of ensuring the nutritional value and amplifying the biological effects of foods [13], okra mucilage has an important functionality. Polysaccharides found in okra mucilage can be an important resource for improving the viability of probiotics used in foods. Depending on the type of food, probiotics will be subject to production steps that are unfavorable to their viability; however, research has shown that probiotic cultures can be significantly protected through encapsulation techniques [86,87].

Rodrigues et al. [88] encapsulated the probiotics *Lactobacillus casei* LC-01 and *L. casei* BGP in sodium alginate microspheres composed of linseed and okra mucilages, botryosphaeran, and commercial fructo-oligosaccharides (FOS) by the extrusion technique in calcium chloride. Laurenti and Garcia [89] used okra mucilage to encapsulate the probiotic *Saccharomyces cerevisiae* by the method of immobilization in agar-agar cubes. The results showed that this mucilage is a natural and alternative encapsulating material and is more efficient than the commercial gums used for this purpose.

## 8. Pharmaceutical Technology

Gums and mucilages may be an alternative in the pharmaceutical industry and have attracted the interest of researchers due to their diverse applications as diluents, binders, disintegrating tablets, thickeners in oral liquids, protective colloids, gelling agents, and suppository bases [40]. They can also be used as a film coating for microencapsulation, administration of osmotic and ophthalmological drugs, oral films, and drug delivery [90].

There is a concern from the pharmaceutical industry for the safety of some synthetic excipients in relation to biological tissues, which influences the reliability of products [91]. Therefore, natural mucilages are preferred over synthetic ones, as they are biocompatible, non-toxic, inexpensive, and easily available [92].

Okra is a great resource for obtaining a safe option of mucilage [93]. According to Nagpal et al. [94], processing okra with a polymer that has the property of forming films, such as chitosan, could increase its use in the production of medicines.

Many polysaccharides are being used as carriers for the delivery of drugs, as they are able to control the rate and release of these substances [95]. As the polysaccharides present in okra mucilage are naturally occurring molecules, they can be an alternative for reducing the side effects of synthetic polymers commonly used by the pharmaceutical industry.

According to Medeiros et al. [96], the combination of two polymers can optimize structural and physical–chemical modifications of the matrix that lead to changes in the characteristics of size, efficiency of encapsulation, speed of release, and, consequently, in the biopharmaceutical properties of the drug. In this sense, Ghumman et al. [97] designed microspheres using okra mucilage combined with alginate for a sustained release of oxcarbazepine and observed significant differences in the pharmacokinetic parameters of the formulation when compared to the pure drug. 

Palei, Mamidi, and Rajangam [98] prepared lamivudine controlled release tablets using different concentrations of okra mucilage as an excipient and observed that the in vitro release decreased with the increasing mucilage concentration, thus confirming its ability to control the release of lamivudine from the matrix. Table 2 shows other applications of okra mucilage as an excipient in pharmaceutical preparations.

From a pharmaco-technical point of view, okra mucilage can be an excellent excipient, increasing viscosity and adding functional properties to cosmetics. Mane et al. [111] concluded that okra mucilage is an alternative to enrich the formulation of hair products, since it is an accessible source of proteins, carbohydrates, minerals, and vitamins.

## 9. Development of Materials

The development of edible films for food packaging is a subject that provokes the interest of researchers to improve mechanical properties, sensory perceptions, convenience, and microbial protection, in addition to extending the useful life of various food products [112]. The production of ecological and sustainable materials encourages the search for natural and low-cost raw materials, such as okra mucilage.

Biodegradable and bioactive edible films represent environmentally friendly food packaging, as they are produced using natural polymeric ingredients, such as sodium alginate, sodium carboxymethylcellulose, and collagen [113].

Natural polysaccharides are an excellent, fully biodegradable and generally inexpensive source of components capable of forming films [114]. Within this aspect, okra mucilage can contribute to the strengthening of the biofilm structure, making it more resistant to traction and improving properties such as the water vapor barrier when compared to films made only with corn starch [115].

Mohammadi et al. [116] evaluated the effect of films based on carboxymethylcellulose, okra mucilage, and ZnO nanoparticles on the shelf life of chicken breast meat stored at 4 °C. The results showed a significant inhibition in microbial growth by the okra mucilage and by ZnO nanoparticles. According to the authors, the two main components of the lipid fraction of okra extract, mainly palmitic and stearic acids, obtained by the addition of acetone during the extraction, were considered responsible for the antimicrobial properties.

According to Cotrim, Mottin, and Ayres [117], the solubility of films in water is an important aspect to be considered when designing food packaging. Some applications require insoluble films, for example, in foods with high sensitivity to moisture, such as meat and fish. In other cases, films with high solubility are essential to remove the packaging with a simple wash before consumption. In films produced with okra mucilage, solubility is directly related to the concentration of mucilage.

## 10. Conclusions

Okra showed to be a promising low-cost functional food for inclusion in the diet or to be used as a raw material for ingredient production due to the important nutrients and bioactive compounds in its composition; the use of its mucilage is of particular interest for human health. Plant-extracted mucilages are important, low-cost, and biocompatible natural resources. Okra is a rich source of mucilage, composed mainly of polysaccharides, which provide rheological characteristics for the use of mucilage for various purposes. In addition, these polysaccharides have important functional properties and are a potential alternative in pharmaceutical development, food preparation, water treatment, and material development. Therefore, research on the use of okra mucilage as a technological adjunct, for example, in the pharmaceutical and food fields, is of great importance. Particularly for its use in food due to its technological and bioactive properties, okra mucilage is a suitable alternative for adding value to products in reason of its important characteristics, such as its capacity for improving texture and its positive health benefits, favoring the development of novel functional foods.

## Figures and Tables

**Table 1:** Composition, methods of extraction, and purification of okra polysaccharides.

Part of Vegetable	Monosaccharide Composition	Extraction	Yield (%)	Purification	Molecular Weight (Da)	References
Leaves	D-ara, D-xyl, D-glu, D-man, and D-gal, Gal, and Xyl	Boiling water (1:15, *w*/*v*) for 3 h	13.0–15.2	Chromatography–mass spectrometry	1.9 × 10^5^–1.6 × 10^6^	[16]
Flowers	Gal, Rha, and GalA	Deionized water (1:40, *w*/*v*) at 100 °C for 3 h	15.33	Ion-exchange chromatography	2.741 × 10^5^	[19]
Pods	Glu, Man, Gal, Ara, Xyl, and Fuc	Ultrasonic extractor using distilled water	10.35	Anion-exchange chromatography	1.92 × 10^5^	[44]
Pods	Ara, Gal, Rha, and GalA	Deionized water (1:10, *w*/*w*) at 75 °C for 2 h	1.1	Anion-exchange chromatography	2.99 × 10^6^	[45]
Pods	Rha, GalA, Gal, GlcA, Glu, and Ara	Distilled water (1:20, *w*/*v*) at 100 °C for 4 h	7.9	Anion-exchange chromatography	5.80 × 10^5^	[49]
Leaves	Ara, Gal, Rha, GalA, and Glu	Ultrasonic extractor using distilled water	3.11	High-performance liquid chromatography	26.9 × 10^3^	[61]

Glu: glucose. Man: mannose. Gal: galactose. Ara: arabinose. Xyl: xylose. Fuc: fucose. Rha: rhamnose: GalA: galacturonic acid. GlcA: glucuronic acid.

**Table 2:** Applications of okra mucilage in pharmaceutical preparations.

Matrix	Drug	Reference
Tablet	Naproxen sodium	[74]
Mucoadhesive films	Turmeric extract	[75]
Bioadhesive patches	Verapamil hydrochloride	[76]
Mucoadhesive beads	Glibenclamide	[99]
Tablets	Losartan potassium	[100]
Tablets	Pentoxifylline	[101]
Tablets	Tramadol HCl	[102]
Mucoadhesive films	Zolmitriptan	[103]
Tablet	Ziprasidone HCl	[104]
Microspheres	Sulfasalazine and dexamethasone	[105]
Tablet	Propranolol HCl	[106]
Tablet	Propranolol HCl	[107]
Floating bioadhesive tablet	Ciprofloxacin	[108]
Tablet	Metformin	[109]
Tablet	Ofloxacin	[110]

## Data Availability

Not applicable.
